# Correction: Synthesis, crystal structure and antibacterial studies of dihydropyrimidines and their regioselectively oxidized products

**DOI:** 10.1039/d1ra90082j

**Published:** 2021-02-18

**Authors:** Alakbar E. Huseynzada, Christian Jelsch, Haji Vahid N. Akhundzada, Sarra Soudani, Cherif Ben Nasr, Aygun Israyilova, Filippo Doria, Ulviyya A. Hasanova, Rana F. Khankishiyeva, Mauro Freccero

**Affiliations:** Baku State University, ICRL Z. Khalilov 23 Baku AZ 1148 Azerbaijan alakbar.huseynzada1117@gmail.com; Université de Lorraine, CNRS, CRM2 54000 Nancy France; Institute of Radiation Problems of ANAS B. Vahabzada 9 Baku AZ 1143 Azerbaijan; Laboratoire de Chimie des Matériaux, Faculté des Sciences de Bizerte, Université de Carthage 7021 Zarzouna Tunisia; Department of Molecular Biology and Biotechnology, Baku State University Z. Khalilov 23 Baku AZ 1148 Azerbaijan; Universita di Pavia V.le Taramelli 10 27100 Pavia Italy

## Abstract

Correction for ‘Synthesis, crystal structure and antibacterial studies of dihydropyrimidines and their regioselectively oxidized products’ by Alakbar E. Huseynzada *et al.*, *RSC Adv.*, 2021, **11**, 6312–6329, DOI: 10.1039/D0RA10255E.

The authors regret that the name of one of the authors (Christian Jelsch) was shown incorrectly in the original article. The corrected author list is as shown above.

The Royal Society of Chemistry apologises for these errors and any consequent inconvenience to authors and readers.

## Supplementary Material

